# Genome-Wide Identification, Expression, and Response to *Fusarium* Infection of the *SWEET* Gene Family in Garlic (*Allium sativum* L.)

**DOI:** 10.3390/ijms24087533

**Published:** 2023-04-19

**Authors:** Mikhail A. Filyushin, Olga K. Anisimova, Anna V. Shchennikova, Elena Z. Kochieva

**Affiliations:** Federal Research Center “Fundamentals of Biotechnology” of the Russian Academy of Sciences, 119071 Moscow, Russia

**Keywords:** garlic, *Allium sativum* L., SWEET uniporters, biotic stress, *Fusarium*, gene structure, gene expression

## Abstract

Proteins of the SWEET (Sugar Will Eventually be Exported Transporters) family play an important role in plant development, adaptation, and stress response by functioning as transmembrane uniporters of soluble sugars. However, the information on the SWEET family in the plants of the *Allium* genus, which includes many crop species, is lacking. In this study, we performed a genome-wide analysis of garlic (*Allium sativum* L.) and identified 27 genes putatively encoding clade I–IV SWEET proteins. The promoters of the *A. sativum* (*As*) *SWEET* genes contained hormone- and stress-sensitive elements associated with plant response to phytopathogens. *AsSWEET* genes had distinct expression patterns in garlic organs. The expression levels and dynamics of clade III *AsSWEET3*, *AsSWEET9*, and *AsSWEET11* genes significantly differed between *Fusarium*-resistant and -susceptible garlic cultivars subjected to *F*. *proliferatum* infection, suggesting the role of these genes in the garlic defense against the pathogen. Our results provide insights into the role of SWEET sugar uniporters in *A. sativum* and may be useful for breeding *Fusarium*-resistant *Allium* cultivars.

## 1. Introduction

Carbohydrates are important biomolecules for energy generation and storage in plants and, as such, are involved in plant development and stress response [1]. Furthermore, sugars define the economic value of many crops. Thus, the content and ratio of fructose and glucose in sink organs significantly affect the taste and quality of grape [2] and tomato [3]) fruit, potato tubers [4], and carrot roots [1]).

Glucose, a basic monosaccharide produced by photosynthesis, is used for the synthesis of fructose and sucrose [5]; then, the three sugars are transported from the leaves through the phloem to accumulating organs (flowers, fruit, seeds, and roots), where they are stored (partly as starch) to support organ growth and development [6,7]. To date, three families of sugar transporters have been characterized: monosaccharide transporters (MSTs), sucrose transporters (SUTs), and Sugar Will Eventually be Exported Transporters (SWEETs) [6,8].

Despite their extensive expansion and functional diversity, the *SWEET* genes are considered to be evolutionarily conserved. A study of this gene family in 31 representative plant species demonstrated a dramatic increase in the number of *SWEET* members in higher plants, largely due to tandem and segmental duplication under purifying selection [9]. Structurally, SWEET transporters are unique due to the presence of up to seven transmembrane domains, which may have arisen during the evolutionary duplication of a prokaryotic ancestral gene. In the plant, SWEETs are involved in maintaining a variety of physiological processes such as pollen nutrition, nectar secretion, seed filling, phloem loading, and pathogen feeding [10].

The members of the SWEET family transport mono- and disaccharides through the plasma membrane in both directions, thus mediating the uptake as well as the efflux of soluble sugars in plant tissues [11,12]. According to their sugar specificity, SWEETs are subdivided into four clades: clades I and II transport hexoses, and clades III and IV—sucrose and fructose, respectively [13,14,15]. SWEETs are known to have organ-specific expression, which, together with their distinct substrate specificity, accounts for diverse physiological functions illustrated by studies in a model species, *Arabidopsis thaliana* L. [16,17,18,19,20,21,22,23,24]. Thus, AtSWEET1 and 2 transport glucose in the rhizosphere and contribute to pathogen resistance. AtSWEET4–8 and 13 also transfer glucose; in addition, AtSWEET5 transports galactose, for which it has dose-dependent sensitivity during pollen germination, whereas AtSWEET8 along with AtSWEET13 is involved in sucrose transport associated with pollen fertility. Four other AtSWEETs are also specific for sucrose: AtSWEET9 participates in sucrose secretion from the nectary parenchyma into the extracellular space, where it is hydrolyzed to form a mixture of sucrose, glucose, and fructose; whereas AtSWEET11, 12, and 15 provide sucrose outflow from the seed coat to the embryo. AtSWEET16 and 17 are associated with fructose transport in leaf and root tonoplasts, where their expression is sensitive to drought. In tomato (*Solanum lycopersicum* L.), the *SlSWEET1a* gene is linked to the *Fgr* (*fructose to glucose ratio*) locus modulating the partitioning of hexoses in the fruit [25,26,27].

Accumulating evidence indicates that SWEETs play a key role not only in sugar transfer, signaling, and plant growth but also in stress tolerance and plant-pathogen interactions [13,28,29]. Many of the 29 tomato *SWEET* genes showed sensitivity to exogenous fructose and sucrose treatment, as well as to high and low-temperature stresses [21]. In *Medicago truncatula*, a large number of 25 *SWEET* genes were found to be upregulated in response to cold, drought, and salt [30]. It has been shown that variations in *SWEET* gene expression can affect resistance to infection through changes in sugar efflux and distribution [29,31,32]. Plant pathogens can release effectors that divert *SWEET* gene expression to benefit infection. Thus, *Xanthomonas oryzae*, the causal agent of bacterial blight, secretes transcription activator-like (TAL) effectors which specifically bind promoters of individual *SWEET* genes in rice, whereas mutations in the effector-binding elements of the *SWEET* promoters confer resistance to blight [13,28,29]. Based on this effect, blight-resistant rice lines have been obtained through *SWEET* promoter editing [31] and gene silencing [32].

The most important sink organ of the plant, the root, imports sugar from the aerial parts of the plant through the phloem and uses it for metabolism and storage, and also releases it into the rhizosphere, where it is assimilated by beneficial microorganisms and pathogens. Sugar transport in all cases is mediated by membrane transporters, including SWEETs [33].

*Fusarium oxysporum*, a root-colonizing fungal pathogen causing root rot, has also been shown to affect *SWEET* gene activity. Thus, inoculation of tobacco (*Nicotiana tabacum* L.) with *F. oxysporum* leads to the downregulation of the *NtSWEET1*, *NtSWEET3b*, and *NtSWEET12* genes and RNAi-mediated suppression of *NtSWEET1* makes tobacco roots more susceptible to rot, indicating the role of NtSWEET1 in tobacco anti-fungal resistance [34]. It has been reported that in sweet potato (*Ipomoea batatas* (L.) Lam.), *F. oxysporum* infection significantly upregulates the *IbSWEET10* gene and that its overexpression or silencing confers resistance or susceptibility to *F. oxysporum*, respectively [35]. In watermelon (*Citrullus lanatus* Thunb.), *ClaSWEET* genes are involved in the resistance to *F. oxysporum* as well as in the response to drought, salt, and cold stresses [36]. Infection of potato (*Solanum tuberosum* L.) with *Fusarium solani*, a necrotroph, or *F. oxysporum* f. sp. *tuberose*, a hemibiotrophic, induces *StSWEET7a* while suppressing most other *StSWEET* genes, and *StSWEET7a* overexpression in potato roots promotes their colonization by *F. oxysporum* f. sp. *tuberosi* through an increase in the sink strength [37]. It is speculated that clade III *SWEET* genes are involved in the biotrophic phase of host-pathogen interaction [38,39]; thus, these genes are upregulated by hemibiotrophic bacteria (*Xanthomonas* or *Pseudomonas*) or fungi (*Golovinomyces cichoracearum*) in rice (*OsSWEET11* and *OsSWEET14*) [28], cassava (*MeSWEET10a*) [38], and *Arabidopsis* (*AtSWEET10*, *AtSWEET12*, and *AtSWEET15*) [16]. At the same time, clade II *SWEET* genes are suggested to play a role in promoting pathogen virulence (*AtSWEET4*, *AtSWEET5*, *AtSWEET7*, and *AtSWEET8* in *Arabidopsis* [16]) or be involved in plant response to necrotrophs (*SWEET4* in *Arabidopsis* and grape [39,40,41]).

Garlic (*Allium sativum* L.) is the second most popular spice and the second most important crop in the *Allium* genus [42,43], with production of over 30 million tons per year (http://www.fao.org/; accessed on 26 January 2023). Garlic is known to have health-promoting and anti-fungal properties, producing compounds that can regulate glucose metabolism and transport and exert therapeutic effects in cerebral ischemia and obesity [44,45] and volatile and non-volatile molecules that act as fungicides [46]. Garlic cloves are rich in carbohydrates, including high-molecular weight fructans and fructooligosaccharides [47,48], as well as soluble sugars such as sucrose, fructose, and glucose [49,50], which constitute up to 75% of the bulb dry matter. Most sugars are photoassimilate-derived, suggesting the existence of an extensive carbohydrate transportation system; however, the information on sugar transporters in garlic is lacking. In particular, nothing is known about SWEETs, not only in garlic, but also in other *Allium* species.

The aim of the present study was to search for and characterize the *A. sativum* genes belonging to the *SWEET* family and investigate their tissue expression patterns. As garlic is susceptible to *Fusarium* infection known to affect *SWEET* expression, we also examined *SWEET* gene activity in response to *F. proliferatum* attack in garlic cultivars resistant and susceptible to *Fusarium* basal rot (FBR).

## 2. Results

### 2.1. Identification of SWEET Genes in the A. sativum Genome

Twenty-seven sequences of full-length *SWEET* genes were found by in silico analysis of the *A. sativum* cv. Ershuizao genome (PRJNA606385) and transcriptome (PRJNA607255) were annotated as *A. sativum* (*As*) *SWEET1–27* (Table 1). Given the low homology between the genes of garlic and the model species *A. thaliana*, combined with the lack of data on *SWEET* genes in *Allium* species, the gene numbering was based on the order of their location on the chromosomes, as was done, for example, for maize *Zea mays SWEETs* [51].

**Figure 1:**
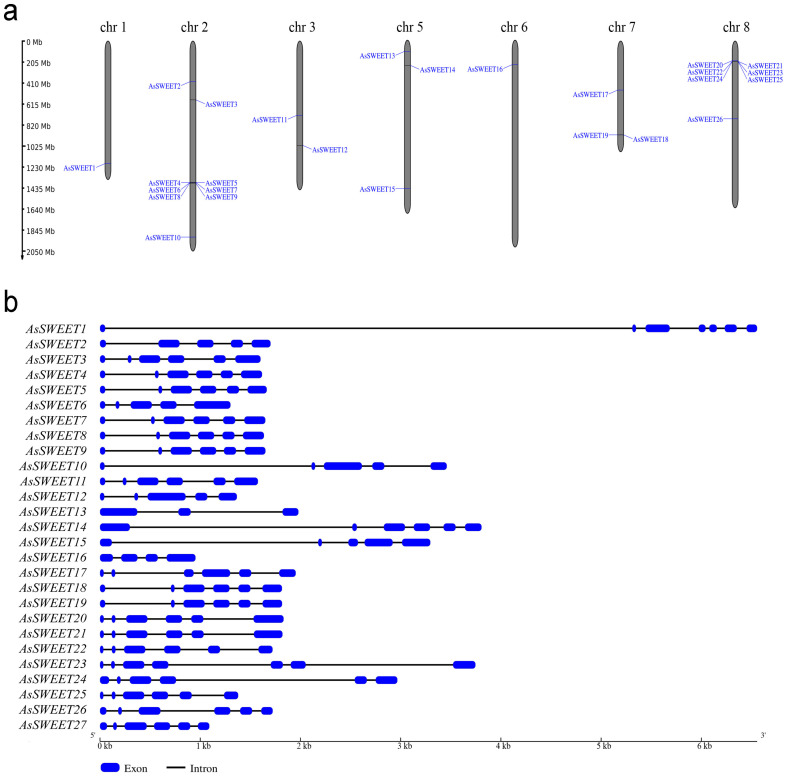
Location and structure of the identified *AsSWEET* genes. (**a**) Chromosomal distribution of the *AsSWEET1–26* genes; *AsSWEET27* is absent as a scaffold-localized gene. The scale on the left indicates chromosome size according to the *A. sativum* cv. Ershuizao genome (PRJNA606385, assembly Garlic.V2.fa) [52]; chr, chromosome; Mb, megabase. (**b**) Predicted exon–intron structures of the *AsSWEET1–27* genes.

The chromosome distribution of the 27 *SWEET* genes was not random: most of them (16) were located on chromosomes 2 (*AsSWEET2*, *AsSWEET3*, *AsSWEET10*, and *AsSWEET4–9* cluster) and 8 (*AsSWEET26* and *AsSWEET20–25* cluster). Ten genes were scattered among chromosomes 1, 3, and 5–7; two of the three genes located on chromosome 7 (*AsSWEET18* and *AsSWEET19*) were tandemly clustered (Figure 1a). The *AsSWEET27* gene was found in scaffold and did not match any chromosome (assembly Garlic.V2.fa; Table 1).

The *AsSWEET* genes significantly varied in size, ranging from 953 bp (*AsSWEET16*) to 6556 bp (*AsSWEET1*), and in the number of exons (3–7) and introns (2–6): most genes (18; ~66.7%) contained 6 exons/5 introns, and 2, 5, 1, and 1 genes contained 7, 5, 4, and 1 exons, respectively. No splice variants were found. The coding sequences (CDSs) varied from 654 bp (*AsSWEET13*) to 1002 bp (*AsSWEET14*) and consisted of 3 (*AsSWEET13*) to 7 (*AsSWEET1* and *AsSWEET23*) exons (Table 1, Figure 1b). An additional search of the transcriptome data for *AsSWEET* mRNA isoforms revealed only one transcript variant for each of the 27 genes.

### 2.2. Characterization of Putative SWEET Proteins in Garlic

The AsSWEET proteins predicted based on the CDSs consisted of 217–333 amino acids (Table 1). Analysis of AsSWEET physicochemical properties indicated that the proteins differed in isoelectric points (pIs) (from 5.84 in AsSWEET16 to 10.01 in AsSWEET21), hydrophobicity index (GRAVY) (from 0.203 in AsSWEET14 to 0.860 in AsSWEET12), and molecular weight (MW) (from 23.991 kDa in AsSWEET13 to 34.077 kDa in AsSWEET23) (Table 1).

**Figure 2:**
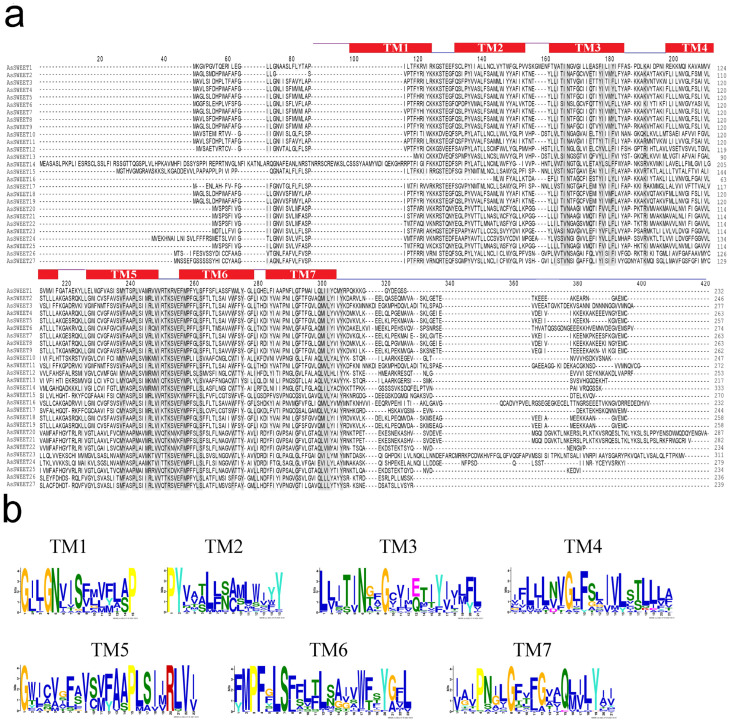
Structure of the predicted AsSWEET proteins. (**a**) Sequence alignment. Gray-shaded regions are 70–100% identical; red blocks on the top indicate positions of transmembrane (TM) helices 1–7. (**b**) Consensus sequences of TM helices 1–7.

The secondary structures of AsSWEETs were characterized by the presence of two MtN3_slv functional domains typical for sugar efflux transporters and several transmembrane helices: seven in most proteins (77.8%), six in AsSWEET13–15, AsSWEET23, and AsSWEET24, and five in AsSWEET16 (Figure 2a), with corresponding consensuses (Figure 2b). In terms of Gene Ontology (GO), all AsSWEET proteins were predicted to have sugar transmembrane transporter activity and be involved in carbohydrate transport.

**Figure 3:**
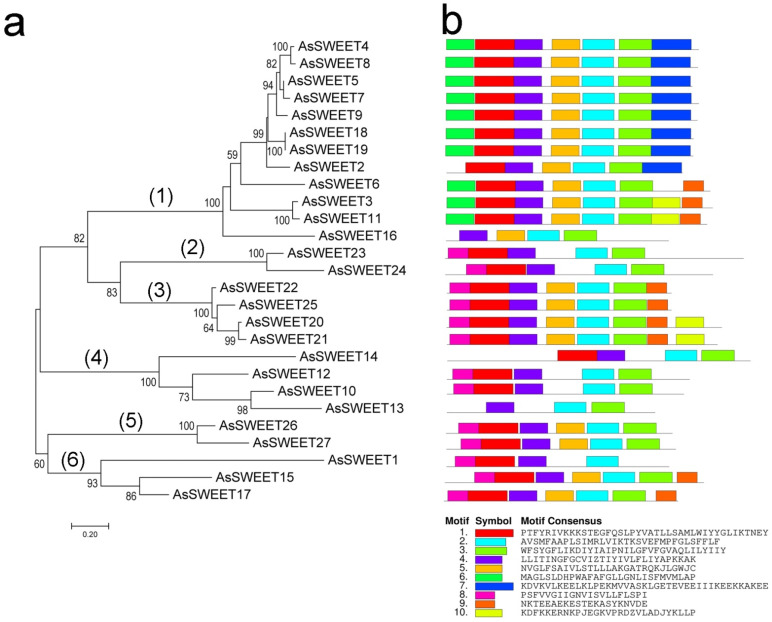
Phylogenetic analysis of the AsSWEET proteins. (**a**) The unrooted dendrogram based on amino acid sequences was constructed using the neighbor-joining method in MEGA7.0.26 [53]. Percentages of replicate trees in which the associated sequences clustered together in the bootstrap test (1000 replicates) are shown next to the branches. (**b**) The distribution of conserved motifs was revealed using MEME 5.4.1. The length of each box is proportional to the size of the motif.

Phylogenetic analysis of AsSWEETs revealed six groups comprising 12 (group 1), 2 (AsSWEET23, AsSWEET24; group 2), 2 (AsSWEET20–22, 25; group 3), 4 (AsSWEET10, AsSWEET12–14; group 4), 2 (AsSWEET26, AsSWEET27; group 5), and 3 (AsSWEET1, AsSWEET15, AsSWEET17; group 6) proteins (Figure 3a). The group division was supported by MEME-based analysis, which revealed 10 conserved motifs (Figure 3b). Among them, motifs 2 and 4 were common for all AsSWEETs, and motifs 1, 3, and 5 were present in most of them with few exceptions: motif 1 was absent in AsSWEET13 and AsSWEET16; motif 2, in AsSWEET1; and motif 5, in AsSWEETs of groups 2 and 4. Motifs 6 and 7 were characteristic for group 1, being present in 10 (except AsSWEET2 and 16) and 8 (except AsSWEET3, AsSWEET6, AsSWEET11, and AsSWEET16) proteins, respectively. In contrast, motif 8 was absent in the AsSWEETs of group 1 but present in those of the other groups (except for AsSWEET13 and AsSWEET14). Motif 9 was found in all AsSWEETs of group 3 and in some of groups 1 and 6, and motif 10, in two members of groups 1 and 3, respectively (Figure 3b).

Thus, besides three tandemly organized *AsSWEET* gene clusters on chromosomes 2, 8, and 7 (Figure 1a), we could also distinguish five phylogenetic clusters (AsSWEET26, AsSWEET27; AsSWEET14, AsSWEET12, AsSWEET10, AsSWEET13; AsSWEET1, AsSWEET15, AsSWEET17; AsSWEET18, AsSWEET19, AsSWEET2; and AsSWEET6, AsSWEET3, AsSWEET11) (Figure 3), the members of which were located on different chromosomes and might have been originated as a result of segmental/transposed gene duplication events.

**Figure 4:**
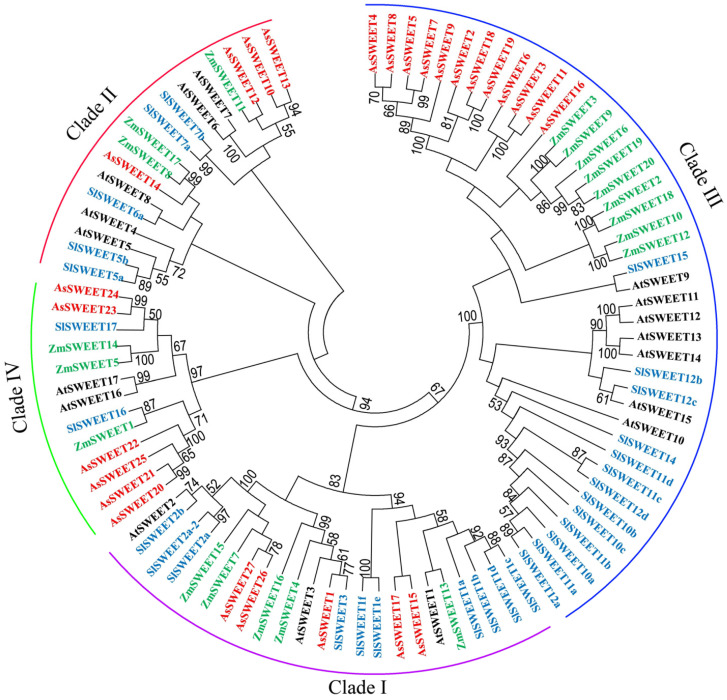
Phylogenetic relationships of SWEET proteins from *A. sativum* (red) and *A. thaliana* (black). Analysis was performed using the neighbor-joining method in MEGA7.0.26 [53]. Percentages of replicate trees in which the associated sequences clustered together in the bootstrap test (1000 replicates) are shown next to the branches. Clades I–IV refer to SWEET clades previously identified in *A. thaliana* [16]. The numbering of maize genes is given in accordance with [51].

Next, we analyzed the relationships of the predicted AsSWEETs and the SWEETs of *A. thaliana*, *S. lycopersicum*, and *Z. mays* since *Arabidopsis*, tomato, and maize are considered models for studying dicot and monocot species, respectively (1) and there are no data on *SWEET* genes in monocot *Allium* species for comparison (2). The results indicated that AsSWEETs of groups 5/6, 4, 1, and 2/3 (Figure 3a) corresponded to *A. thaliana* SWEET clades I, II, III, and IV, respectively, and that the tandemly organized *AsSWEET* gene clusters (Figure 1a) corresponded to specific functional *A. thaliana* SWEET clades: *AsSWEET4–9*, *AsSWEET18*, and *AsSWEET19*, to clade III and *AsSWEET20–25*, to clade IV (Figure 4).

It was found that, within clades I, II, and IV, the garlic genes split into subclades based on similarity to genes of *Arabidopsis*, tomato (dicot), and maize (monocot). In clade I, AsSWEET1 was grouped with At/SlSWEET3 and ZmSWETT4/ZmSWETT16, AsSWEET26/AsSWEET27 with At/SlSWEET2 and ZmSWETT15/ZmSWETT17; and AsSWEET15/AsSWEET17 with At/SlSWEET1 and ZmSWETT13. In clade II, AsSWEET10/AsSWEET12/AsSWEET13 formed subclade with At/SlSWEET6, AtSWEET7 and ZmSWETT11; and AsSWEET14 formed subclade with At/SlSWEET5, AtSWEET4, AtSWEET8, SlSWEET16a, and ZmSWETT17/ZmSWEET18. In clade IV, AsSWEET23/AsSWEET24 clustered with AtSWEET16, At/SlSWEET17, and ZmSWETT14/ZmSWEET15; and AsSWEET20–22 and AsSWEET25 with SlSWEET16 and ZmSWETT1 (Figure 4).

Clade III had the largest number of members, but the SWEETs of garlic, as well as maize, were grouped predominantly separately from the SWEET proteins of dicotyledonous plant species. All AsSWEETs (AsSWEET4–9, AsSWEET18, and AsSWEET19) are included in the *Z. mays* ZmSWEET3, ZmSWEET6, ZmSWEET9, ZmSWEET19, and ZmSWEET320 subclade (Figure 4). Similar clustering of clade III proteins, preferentially separating monocot and dicot proteins, is shown, for example, in [54].

In addition, NCBI-BLAST was used to determine the closest homologs of AsSWEET proteins in two model plant species, *A. thaliana* and *S. lycopersicum* (Table S1). It was found that the proteins of garlic have only 49–67% identity with the SWEETs of tomato and *Arabidopsis*. Clade III AsSWEETs were the most similar to AtSWEET11 and AtSWEET12 (except for AsSWEET16, which was close to AtSWEET14) in *A. thaliana* and to SlSWEET10c, SlSWEET12b, SlSWEET11c in *S. lycopersicum*. Clade I proteins were the closest homologs of AtSWEET3/SlSWEET3 (AsSWEET1), AtSWEET1/SlSWEET1b (AsSWEET15 and AsSWEET17) and AtSWEET2/SlSWEET2a-2 (AsSWEET26 and AsSWEET27). The highest similarity of clade II and IV proteins was observed with AtSWEET7/SlSWEET7a and AtSWEET17/SlSWEET16, respectively (Supplementary Table S1).

### 2.3. Cis-Acting Elements in the AsSWEET Promoters

**Figure 5:**
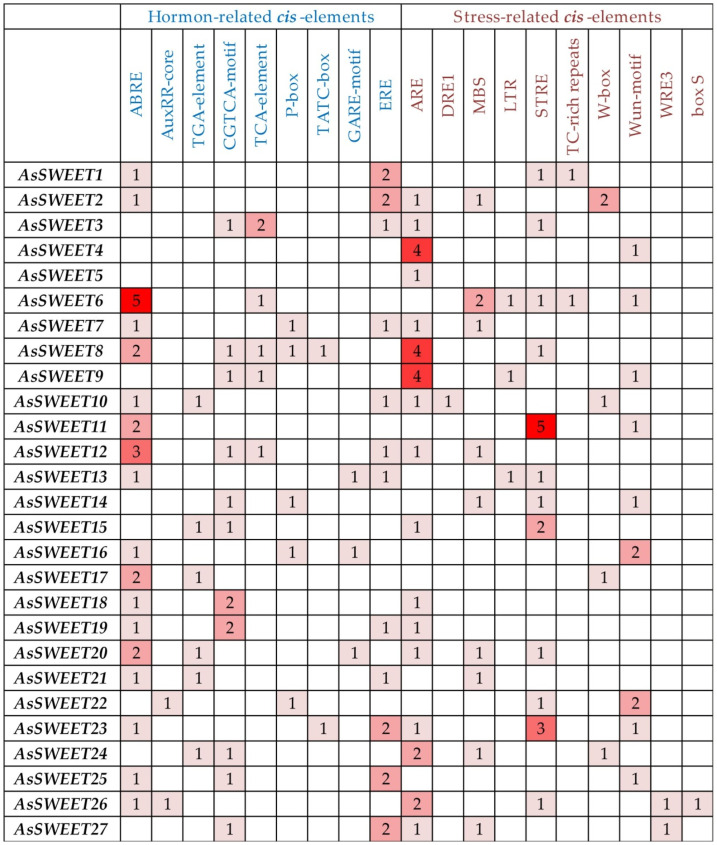
Hormone- and stress-related cis-acting elements found in the promoter regions of AsSWEET genes. MeJA, methyl jasmonate. The color scheme (from pale to dark) corresponds to the numbers of *cis*˗elements (from low to high).

Considering the role of SWEETs in plant stress- and hormone-related responses [14,15,16,17,18,19,20,21,22], we searched the *AsSWEET* promoter regions (~1 kb upstream of the initiation codon) for the stress- and hormone-sensitive *cis*-regulatory elements. The results showed that the promoters of the *AsSWEET6*, *AsSWEET8*, and *AsSWEET23* genes were the most enriched in *cis*-elements, containing 12, 11, and 9 of them, respectively (Figure 5).

Nine types of hormone-responsive *cis*-elements, including those involved in the response to abscisic acid (ABA), auxin (AuxRR), methyl JA (MeJA), salicylic acid (SA), gibberellic acid (GA), and ethylene (ET), were identified in *AsSWEET* genes (Figure 5). Each *AsSWEET* promoter had 2–5 elements, except for *AsSWEET4* and *5*, which had none. The largest number of one-type hormone-sensitive elements (ABA-responsive) was detected in *AsSWEET6* (clade III) (Figure 5).

In total, 10 types of *cis*-elements related to stresses such as anaerobic conditions, drought, cold, wounding, and pathogens were identified in *AsSWEET* promoters, all of which contained at least one (Figure 5). Two genes, *AsSWEET4* and *AsSWEET5*, contained only stress-related elements; the former, along with *AsSWEET8* and *AsSWEET9*, had the largest number (4) of elements responsive to anaerobic conditions. The largest number of STREs was found in *AsSWEET8* (5), followed by *AsSWEET23* (3). *AsSWEET16* and *AsSWEET17* contained only wound-responsive elements—two Wun-motifs and one W-box, respectively; the other wounding/pathogen-responsive motifs (WRE3 and/or box S) were found only in *AsSWEET26* and *27*. A drought-responsive element was identified only in *AsSWEET10* (Figure 5).

### 2.4. Tissue Expression Patterns of AsSWEET Genes

**Figure 6:**
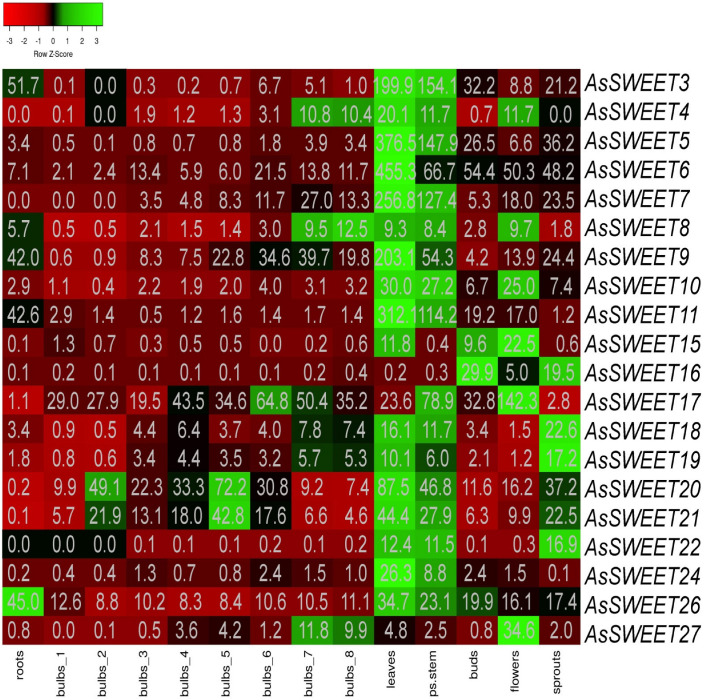
Heatmap of *AsSWEET* expression in *A. sativum* cv. Ershuizao (PRJNA607255). *AsSWEET* mRNA levels were analyzed in the roots, bulbs (stages 1–8 corresponding to 192-, 197-, 202-, 207-, 212-, 217-, 222-, and 227-day-old bulbs, respectively), leaves, pseudostems (ps.stem), buds, flowers, and sprouts. The color scheme indicates gene expression gradient from low (red) to high (green).

Analysis of the *A. sativum* cv. Ershuizao transcriptome (PRJNA607255) revealed that seven *AsSWEET genes* (*AsSWEET1*, *AsSWEET*2**, *AsSWEET*12**–*14*, *AsSWEET23*, and *AsSWEET25*) were not transcribed in garlic. The majority of the transcribed genes had the highest mRNA levels in the leaves, except for *AsSWEET16* (maximum in the buds), *AsSWEET15*, *AsSWEET17*, *AsSWEET27* (flowers), *AsSWEET18*, *AsSWEET19*, *AsSWEET22* (sprouts), and *AsSWEET26* (roots). High levels of *AsSWEET* expression were also detected in the pseudostem (except for *AsSWEET15*, *AsSWEET16*, and *AsSWEET27*). *AsSWEET3*, *AsSWEET4*, *AsSWEET7*, *AsSWEET22*, and *AsSWEET27* were not expressed in the stage 1/2 bulbs, whereas *AsSWEET4*, *AsSWEET7*, *AsSWEET15–17*, and *AsSWEET20–24* had very low or no expression in the roots (Figure 6).

Organ-specific expression was observed for the following AsSWEET genes: AsSWEET3, AsSWEET9, AsSWEET11, and AsSWEET26 (roots), AsSWEET3, AsSWEET5–7, AsSWEET9, AsSWEET16, AsSWEET18–22, and AsSWEET26 (sprouts), AsSWEET6, AsSWEET10, AsSWEET15, AsSWEET17, and AsSWEET27 (flowers), AsSWEET3, AsSWEET5, AsSWEET6, AsSWEET16, AsSWEET17, and AsSWEET26 (buds), AsSWEET17, AsSWEET20, AsSWEET21, and AsSWEET26 (bulbs of all stages), and AsSWEET4, AsSWEET7–9, and AsSWEET27 (bulbs at the final [6,7,8] stages) (Figure 6). These data indicated that genes of each SWEET clade were expressed in the analyzed organs. Given the previously shown role of SWEETs in the transport of hexoses (clades I and II), sucrose (clade III), and fructose (clade IV) [11,12,13], we may assume that AsSWEETs of clades I, II, and IV are involved in the transport of both hexoses and clade III AsSWEETs—in the sucrose transport.

Tissue expression patterns differed for the genes of two chromosomal clusters (*AsSWEET4–9* and *AsSWEET20–25*), whereas those of the third cluster genes (*AsSWEET18*, *AsSWEET19*) were similar. Presumably segmentally duplicated genes (*AsSWEET26*, *AsSWEET27*; *AsSWEET11*, *AsSWEET15*, *AsSWEET17*; and *AsSWEET6*, *AsSWEET3*, *AsSWEET11*) also showed different tissue expression patterns. In phylogenetic group 4, only *AsSWEET10* was expressed; in group 1, *AsSWEET2*, as a possible segmentally duplicated variant of *AsSWEET18* and *AsSWEET19*, was not transcribed (Figure 6).

**Figure 7:**
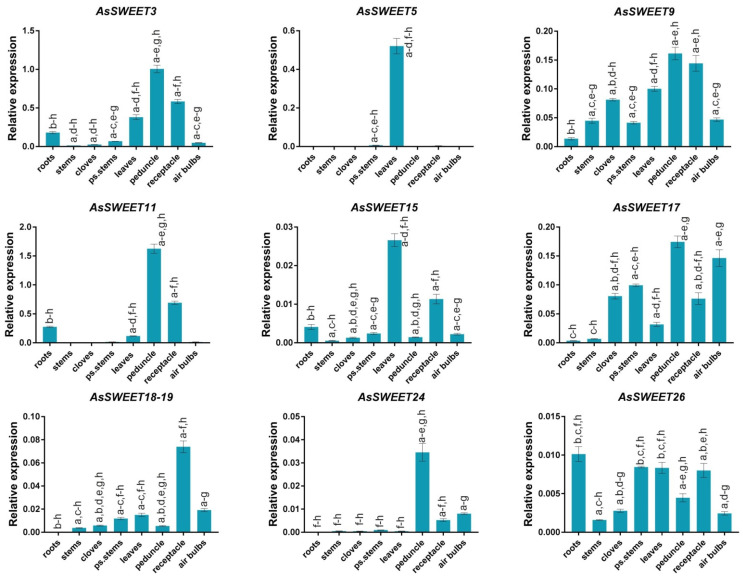
Transcription of the selected *AsSWEET* genes in *A. sativum* cv. Sarmat tissues. “*AsSWEET18–19*” means the sum expression of *AsSWEET18* and *AsSWEET19* genes; the high identity (over 98%) of their mRNAs forced the design of common primers. The data were normalized to glyceraldehyde 3-phosphate dehydrogenase (*GAPDH*) and ubiquitin (*UBQ*) mRNA levels; ^a–h^ *p* < 0.01 indicates significant differences between tissues; ps.stems, pseudostems.

The expression of 11 genes (*AsSWEET1*, *AsSWEET15*, *AsSWEET17*, and *AsSWEET26* of clade I, *AsSWEET13* of clade II, *AsSWEET3*, *AsSWEET5*, *AsSWEET9*, *AsSWEET11*, and *AsSWEET18*/*19* of clade III, and *AsSWEET24* of clade IV) was verified by quantitative real-time (qRT)-PCR in the tissues of *A. sativum* cv. Sarmat (Figure 7). It was confirmed that *AsSWEET1* and *AsSWEET13* were not transcribed in any garlic tissue. The results also revealed that the expression of most analyzed genes in the roots, cloves, leaves, and pseudostems was similar in cv. Ershuizao and Sarmat (Figure 6 and Figure 7); however, three genes showed different expression levels: *AsSWEET5* in the pseudostem (high/traces, respectively), *AsSWEET15* in the roots (traces/high, respectively), and *AsSWEET11* and *AsSWEET24* in the leaves and pseudostems (high/low, respectively).

*AsSWEET3*, *AsSWEET9*, *AsSWEET15*, *AsSWEET17*, and *AsSWEET26* were expressed in all analyzed tissues with the maximal level in the roots (*AsSWEET26*), leaves (*AsSWEET5* and *AsSWEET15*), peduncles (*AsSWEET3*, *AsSWEET9*, *AsSWEET11*, *AsSWEET17*, and *AsSWEET24*), and receptacles (*AsSWEET9* and *AsSWEET18–19*). *AsSWEET5* was expressed only in the leaves. The expression of *AsSWEET9* and *AsSWEET17* was characteristic for the cloves and air bulbs, whereas that of *AsSWEET5* and *AsSWEET11* was absent in these organs, and that of *AsSWEET5*, *AsSWEET18–19*, and *AsSWEET24* was absent in the roots (Figure 7).

### 2.5. AsSWEET Gene Expression in Response to F. proliferatum Infection

Considering that SWEETs are involved in plant resistance to *Fusarium* spp. [34], the expression of the genes which showed differential tissue expression patterns (*AsSWEET3*, *AsSWEET5*, *AsSWEET9*, *AsSWEET11*, *AsSWEET18*, and *AsSWEET19* [clade III], *AsSWEET15*, *AsSWEET17*, and *AsSWEET26* [clade I], and *AsSWEET24* [clade IV]) (Figure 7) was compared in the roots of garlic cultivars resistant (cv. Sarmat) and susceptible (cv. Strelets) to FBR. The plants were infected with *F. proliferatum* and analyzed 24 and 96 h post-inoculation (hpi), covering the peak of pathogenesis-related *(PR)* gene expression in response to hemibiotrophic pathogens [55,56,57,58].

**Figure 8:**
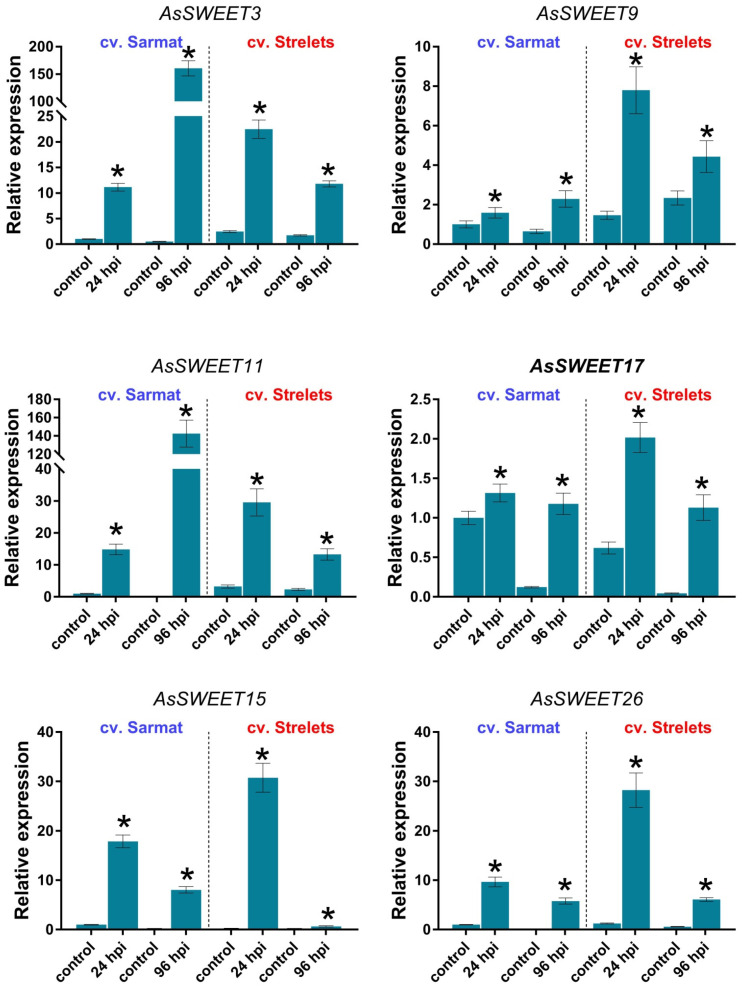
Expression of the selected *AsSWEET* genes in the roots of *A. sativum* FBR-resistant cv. Sarmat and FBR-susceptible Strelets in response to *F. proliferatum* infection. The plants were incubated with *F. proliferatum* conidia and analyzed for the transcription of the indicated genes at 24 and 96 h post-inoculation (hpi). The data were normalized to *GAPDH* and *UBQ* mRNA levels and presented as fold change (mean ± SE) of control (24 h in cv. Sarmat taken as 1); * *p* < 0.01 compared to uninfected control.

The expression of *AsSWEET5*, *AsSWEET18*, *AsSWEET19*, and *AsSWEET24* was not detected either in non-infected or infected roots, which is consistent with the previous results (Figure 7), whereas that of the other six genes (*AsSWEET3*, *AsSWEET*9, *AsSWEET11*, *AsSWEET15*, *AsSWEET17*, and *AsSWEET26*) was affected by *F. proliferatum* infection (Figure 8). At 24 hpi, *AsSWEET3*, *AsSWEET11*, *AsSWEET15*, and *AsSWEET26* were significantly upregulated in both cultivars, especially in cv. Strelets, where *AsSWEET9* was induced about 8-fold, *AsSWEET3*—over 20-fold, and *AsSWEET11*, *AsSWEET15*, and *AsSWEET26*—about 30-fold compared to the uninfected control (Figure 8). Thus, the *AsSWEET* genes were strongly upregulated in the FBR-susceptible cultivar at the early stage of *Fusarium* infection. Later (96 hpi), the expression level of all *AsSWEET* genes in cv. Strelets decreased but still remained significantly higher than in control; at the same time, in cv. Sarmat, the expression of *AsSWEET3* and *AsSWEET11* was increased 140–150 times (Figure 8). These results indicated cultivar-dependent activation of *AsSWEET* genes in response to *Fusarium* infection.

### 2.6. Effect of F. proliferatum Infection on Sucrose, Glucose, and Fructose Contents in Garlic

**Figure 9:**
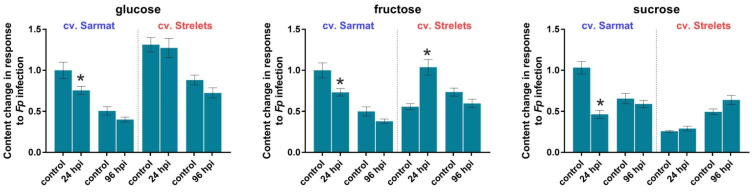
Changes in glucose, fructose, and sucrose contents in the roots of FBR-resistant cv. Sarmat and FBR-susceptible cv. Strelets in response to *F. proliferatum* infection. The plants were incubated with *F. proliferatum* conidia and analyzed for sugar contents at 24 and 96 h post-inoculation (hpi). The data are presented as fold change (mean ± SE) of control (24 h in cv. Sarmat taken as 1); * *p* < 0.01 compared to uninfected control.

Next, we compared the content of soluble sugars (glucose, fructose, and sucrose) in infected and non-infected garlic cultivars. The results indicated that at 24 hpi, the levels of all sugars were significantly decreased in the roots of FBR-resistant cv. Sarmat but not in those of FBR-susceptible cv. Strelets where the levels of glucose and sucrose were unchanged and that of fructose significantly increased (Figure 9). No significant changes in the sugar content were observed in both cultivars at 96 hpi. This may indicate that the FBR-resistant cv. Sarmat does not supply *Fusarium* with sugars, while the FBR-sensitive cv. Strelets supplies pathogen in an early response to infection but may stop efflux at 96 hpi.

### 2.7. Cloning and Characterization of CDSs and Regulatory Regions of AsSWEET Genes Differentially Expressed in FBR-Sensitive and -Resistant Cultivars after F. proliferatum Infection

Considering the differential expression of *AsSWEET3*, *AsSWEET9*, *AsSWEET11*, and *AsSWEET26* genes in cv. Sarmat and Strelets in response to *F. proliferatum* infection (Figure 8), we cloned and sequenced the CDSs and regulatory regions of these genes and compared them with those of cv. Ershuizao (used as reference).

The results indicated that each cloned CDS had 2–5 single nucleotide polymorphisms (SNPs) (Table 2). Fifteen SNPs were shared by cv. Sarmat and Strelets; among them, only one (c.293A>G in *AsSWEET3*) was non-synonymous and led to amino acid substitution p. K98R (Table 2). In cv. Sarmat, the *ASWEET3* gene contained an additional non-synonymous SNP (c. 758T>C leading to p. V253A). The other cultivar-specific polymorphisms were found in *AsSWEET11*, which in cv. Sarmat contained a non-synonymous SNP c.734A>T (p. E245V) and in cv. Strelets—synonymous c. 69C>T and non-synonymous c. 761T>C (p. I254T) SNPs (Table 2).

Analysis of the regulatory regions (~1 kb, including promoter; ~0.7 kb in case of *AsSWEET11*) indicated that those of *AsSWEET3* had a 340 bp deletion and 3 SNPs in both cv. Sarmat and Strelets; in addition, this gene had a 3 bp insertion in cv. Sarmat and carried 2 SNPs in cv. Strelets. In both cv. Sarmat and Strelets, the promoter regions of *AsSWEET9* had a 2 bp deletion and 12 SNPs, those of *AsSWEET11*–2 SNPs, and those of *AsSWEET26*–8 SNPs. In addition, the *AsSWEET26* promoter of cv. Sarmat carried four extra SNPs and a 10 bp deletion, whereas that of cv. Strelets had 10 extra SNPs (Table 2).

The identified polymorphisms led to changes in the composition of *cis-*regulatory elements (Table 3). Thus, the *AsSWEET3* promoter in both cultivars lacked the F-box element and that in cv. Strelets contained the WRE3 element. In both cv. Sarmat and Strelets, the promoter of the *AsSWEET9* gene acquired box 4 but lost several AE-boxes, that of *AsSWEET11* lost a STRE, and that of *AsSWEET26* acquired TGA, CGTCA, and P-box elements and extra MYC elements but lost some ARE and MYB elements; furthermore, in cv. Sarmat, the *AsSWEET9* promoter lacked the AuxRR-core element.

## 3. Discussion

SWEET uniporters that mediate the bidirectional transfer of soluble sugars between different tissues are important not only for plant growth and development but also for the immune response [13,28,29,31,32].

Accumulating evidence indicates that during infection, plant pathogens selectively target SWEET transporters to access host carbohydrate reserves and obtain soluble sugars [39]. Thus, *Xanthomonas* spp. are known to upregulate the expression of specific *SWEET* genes; conversely, the blockage of *SWEET* activation can increase plant resistance to infection and reduce pathogen growth and virulence [39]. It is suggested that the suppression of *SWEET* genes in plants can reduce their sensitivity to infection and may potentially be employed as a strategy to breed resistant crop cultivars [31,32,39]. However, this approach is not entirely correct since symbiotic arbuscular mycorrhizal (AM) fungi, which can significantly enhance plant growth and adaptability by colonizing their roots, also obtain carbohydrates from the root cortex apoplast, including through the SWEET activities. For example, colonization of potato roots by the AM fungus *Rhizophagus irregularis* causes significant changes in the transcription of 22 out of 35 genes of the *SWEET* family [59]. Therefore, functional studies of the specific *SWEET* genes can help to determine the balanced effect on the transporter activity, which would contribute to plant protection, on the one hand, and, on the other hand, would not break the symbiotic potential of plant-microbial communities.

The present study was the first attempt to identify and characterize *SWEET* genes in the *Allium* genus in general and in garlic in particular. Overall, we found 27 *SWEET* genes in *A. sativum* cv. Ershuizao through genome-wide analysis (Table 1). Multiple *SWEET* genes are characteristic of many species, including agricultural crops; thus, there are 17 such genes in *A. thaliana*, 25 in *Musa acuminate* Colla, 29 in *S. lycopersicum*, and 108 in *Triticum aestivum* L. [60]. However, if in tomato, for example, the size of gene families can be regulated through sexual reproduction and introgressive hybridization [60,61,62], it is not the case in garlic, which is an asexually reproducing (apomictic) species whose genotypes presumably originate through mutations in a single clone, leading to significant phenotypic diversity [63]. It can be speculated that the high number of *SWEET* genes in garlic is a result of accelerated random mutagenesis and somaclonal variation observed especially in the genes related to the basic processes of plant development and adaptability [60,61,62]. Alternatively, it is possible that the *SWEET* gene family in *A. sativum* had evolved before this species lost the ability for sexual reproduction.

Our structural analysis revealed As*SWEET* genes of all currently known *SWEET* clades (I–IV) (Figure 2, Figure 3 and Figure 4), suggesting functional conservation of the bidirectional transport of soluble sugars: hexoses (clades I and II), fructose (clade IV), and sucrose (clade III) [13,14,15].

The observed clustering of *AsSWEET20–25* (clade IV) and *AsSWEET18/19* and *AsSWEET4–9* (clade III) on chromosomes 8, 7, and 2, respectively (Figure 1a) suggests their evolutionary origin through tandem duplication and points on the functional redundancy of paralogous transporters within these groups. Differences in the expression patterns of *AsSWEET* genes in the three clusters, including complete silencing of some members (Figure 6 and Figure 7), may reflect the neofunctionalization effect of duplicated genes due to mutations during adaptation to environmental changes. Such mutations can occur in the CDSs as well as in the regulatory sequences, as evidenced by the obvious differences in the patterns of *cis*-regulatory elements in the *AsSWEET* promoter regions (Figure 5).

The presence of hormone- and stress-responsive elements in *AsSWEET* promoters (Figure 5) (which is also characteristic of garlic *PR* genes [54,55,56]) is consistent with the involvement of SWEETs in hormone-mediated as well as in hormone-independent signaling pathways regulating plant responses to abiotic and biotic stresses, including *Fusarium* infection [28,29,35,36,37,55,56,57].

FBR of the bulbs and *Fusarium* wilt of the leaves can lead to significant (up to 60%) losses of the garlic crop [64,65,66,67,68,69]. *F. proliferatum*, one of the *Fusarium* species infecting garlic [57], is a common hemibiotrophic pathogen that utilizes plant sugars both in the biotrophic and necrotrophic phases [70]. Therefore, we hypothesized that this fungus could activate carbohydrate metabolism in garlic through the regulation of *AsSWEET* genes. Analysis of *AsSWEET* transcription in the roots of FBR-resistant and -susceptible garlic cultivars at the biotrophic (24 hpi) and pre-necrotrophic (96 hpi) phases of *F. proliferatum* infection revealed the upregulation of six genes, *AsSWEET3*, *AsSWEET9*, *AsSWEET11*, *AsSWEET15*, *AsSWEET17*, and *AsSWEET26*, in response to infection in both cultivars (Figure 8). Among these genes, *AsSWEET3*, *AsSWEET9*, and *AsSWEET11* belong to clade III (Figure 4), the members of which are known to be induced in plants at the biotrophic phase of pathogen infection [39]. However, there was a significant difference in the activation level of *AsSWEET3*, *AsSWEET9*, and *AsSWEET11* between FBR-resistant and -susceptible cultivars both at 24 and 96 hpi (Figure 8), which is consistent with the role of clade III SWEETs in response to *Fusarium* infection. Thus, the expression of these genes was much more significantly upregulated in the sensitive cv. Strelets than in the resistant cv. Sarmat at 24 hpi (Figure 8), suggesting pathogen-induced activation in order to access host sugars and promote colonization at the biotrophic stage. At the same time, *AsSWEET3*, *AsSWEET9*, and *AsSWEET11* were much stronger upregulated in the resistant cv. Sarmat than in the susceptible cv. Strelets at the pre-necrotrophic (96 hpi) stage of infection (Figure 8). It is possible that the late-stage induction of *AsSWEET3*, *AsSWEET9*, and *AsSWEET11* in the resistant cultivar could represent an attempt of the host plant to stimulate sugar outflow from the infected roots in order to restrict pathogen growth, which is in agreement with a decrease in fructose, glucose and sucrose levels in the roots of cv. Sarmat but not in those of cv. Strelets (Figure 9), as well as the fact that sugars act as signaling molecules and interact with the hormonal network that regulates the immune system of plants [71]. Differential expression patterns of *SWEET* genes have also been reported in *Fusarium*-resistant and -susceptible cultivars of watermelon [36]. Thus, our results, together with previous data, indicate distinct roles of individual SWEETs in plant resistance to fungal infection.

The three genes of clade I (*AsSWEET15*, *AsSWEET17*, and *AsSWEET26*) upregulated by *F. proliferatum* infection showed overall similar dynamics and degree of activation in both cultivars (Figure 8), suggesting that the regulatory pathways underlying *AsSWEET15*, *AsSWEET17*, and *AsSWEET26* transcriptional induction in response to infection are not involved in the garlic resistance to *Fusarium*.

Despite the differential transcriptional response to the infection of *AsSWEET3*, *AsSWEET9*, *AsSWEET11*, and *AsSWEET26*, sequence alignment revealed a high degree of structural similarity in their *cis*-regulatory regions and protein products in FBR-susceptible and -resistant garlic cultivars (Table 2 and Table 3). This fact points to intricate molecular mechanisms controlling *SWEET* gene expression in garlic, which are likely genotype-dependent and defines the strength of garlic resistance to *Fusarium* infection.

Earlier studies of FBR-resistant and -susceptible garlic cultivars have identified a set of *A. sativum PR* genes encoding chitinases, glucanases, and thaumatin-like proteins, which also have infection stage-dependent activation patterns and could be involved in anti-fungal immune defense [55,56,57]. Together with these findings, our present results suggest that *AsSWEET* genes can be a part of a complex protection system evolved in garlic to resist biotic as well as abiotic stresses, which requires further investigation.

## 4. Materials and Methods

### 4.1. Identification and Structural Analysis of the A. sativum SWEET Genes

The search for the full-length *SWEET* genes was performed in the *A. sativum* cv. Ershuizao genome (PRJNA606385, assembly Garlic.V2.fa) according to the annotated transcriptome (PRJNA607255) (genome and transcriptome were prepared by Sun et al. (2020) [52]); only sequences with start and stop codons and two MtN3_slv domains (pfam03083) in the translated version were selected. Comparative gene and protein structural analyses were conducted with MEGA 7.0.26 [53]. The phylogenetic dendrogram was constructed based on protein sequences using MEGA 7.0.26 (Neighbor-Joining method); confidence for tree topologies was estimated by bootstrap values of 1000 replicates. Used in analysis *A. thaliana* and *S. lycopersicum* SWEET numbering is given according to [27] and [21,72], respectively.

The chromosomal localization map was drawn using MG2C v. 2.1 (http://mg2c.iask.in/mg2c_v2.1/; accessed on 26 January 2023). To predict exon–intron structures, *AsSWEET* genes, and their CDSs were analyzed using GSDS v2.0 [73]. Putative proteins were characterized by MW, pI, and GRAVY (ExPASy ProtParam; https://web.expasy.org/protparam/; accessed on 26 January 2023), conserved domains (NCBI-CDD, https://www.ncbi.nlm.nih.gov/cdd; accessed on 26 January 2023), consensuses (MEME 5.5.1, http://meme-suite.org/tools/meme; accessed on 26 January 2023), GO biological processes (PANNZER2; http://ekhidna2.biocenter.helsinki.fi/sanspanz/; accessed on 26 January 2023), and transmembrane helices (TMHMM-2.0; https://services.healthtech.dtu.dk/service.php?TMHMM-2.0; accessed on 26 January 2023).

### 4.2. In Silico mRNA Expression Analysis

*AsSWEET* gene expression in garlic tissues was analyzed based on *A. sativum* cv. Ershuizao RNA-seq dataset (ID: PRJNA607255), which was normalized as Fragments Per Kilobase of transcript per million mapped reads (FPKM) [52] and included data on the gene expression profiles in the roots, bulbs (8 developmental stages), leaves, pseudostems, buds, flowers, and sprouts. According to Sun et al. (2020) [52], buds were collected at the bud stage of garlic plants planted in October 2018; samples of roots, leaves, sprouts, pseudostems (212 days after sowing), and flowers (217 days after sowing) were collected in May 2019; samples of bulbs of eight growth stages were taken every five days from April 11 to May 16, 2019. Transcripts with an average FPKM value of ≥10 in at least one tissue type were selected and used for heatmap construction with Heatmapper [74].

### 4.3. Plant and Fungi Material and F. proliferatum Infection

FBR-resistant cv. Sarmat and FBR-susceptible cv. Strelets used in this study are winter garlic cultivars of Russian breeding. Bulbs from the 2022 harvest were kindly provided by the Federal Scientific Vegetable Center (Moscow region, Russia).

The *F. proliferatum* strain was originally isolated from the bulbs of garlic cv. Strelets was kindly provided by the Group of Experimental Mycology, Winogradsky Institute of Microbiology (Research Center of Biotechnology of the RAS, Moscow, Russia). According to the pathogenicity test, the first signs of the disease appear on the clove surface 5 days after infection [55].

*F. proliferatum* infection was performed as previously described [56]. Cloves were surface sterilized in 70% ethanol for 3 min, rinsed with sterile water, placed in Petri dishes with wet filter paper, and incubated at +25 °C in the dark for 72 h until active root growth was observed. Then, cloves were infected by soaking in *F. proliferatum* conidial suspension (~10^6^ conidia mL^−1^) for 5 min, transferred to fresh Petri dishes, and incubated at +25 °C in the dark for 24 and 96 h (*n* = 3 cloves per each time point); uninfected cloves were used as control. The roots, pseudostems, and cloves were collected at each time point, frozen in liquid nitrogen, and stored at −80 °C.

### 4.4. RNA Extraction and qRT-PCR Analysis

Total RNA was extracted from garlic tissues (0.1 g of each) using the RNeasy Plant Mini Kit (QIAGEN, Hilden, Germany), cleaned from genomic DNA (RNase free DNase set; QIAGEN), qualified by gel electrophoresis, and used for first-strand cDNA synthesis (GoScript Reverse Transcription System; Promega, Madison, WI, USA) with an oligo-dT primer. RNA and cDNA concentrations were quantified by fluorimetry (Qubit^®^ Fluorometer, Thermo Fisher Scientific, Waltham, MA, USA). qRT-PCR was performed in a CFX96 Real-Time PCR Detection System (Bio-Rad Laboratories, Hercules, CA, USA) with 3.0 ng cDNA, SYBR Green RT-PCR mixture (Syntol, Moscow, Russia), and specific primers. Primers were designed based on *A. sativum* cv. Ershuizao transcriptomic data (PRJNA607255) (Table S2) as a result of comparative analysis of *AsSWEET* mRNAs; the most variable regions of each gene were used to select primers so that there was at least one intron between them. Further, manual revision of sequence polymorphisms and additional evaluation was performed using Primer3 (http://frodo.wi.mit.edu/primer3/, accessed on 15 October 2022). Primers were experimentally tested at three concentrations of cDNA (mix from various organs)—10, 1, and 0.1 ng per well in three technical replicates. Primer pairs with only one significant peak occurring in the melt curve above the instrument detection limit were selected; the efficiency of the validated pairs of primers was 95–110%, R^2^ = 0.943–0.999. Given the high identity (over 98%) of the *AsSWEET18* and *AsSWEET19* mRNAs, common primers were designed for these genes (Table S2). The following cycling conditions were used: initial denaturation at 95 °C for 5 min and 40 cycles of denaturation at 95 °C for 15 s, and annealing/extension at 60 °C for 40 s.

mRNA expression of *AsSWEET* genes was normalized to that of two reference genes, *GAPDH* and *UBQ* [56], which were previously used separately as a reference in onion (*Allium cepa* L.) and garlic [75,76]. We used the combination of *GAPDH* and *UBQ* based on studies showing that the combination of two stably expressed reference genes, rather than one, improves the accuracy of qPCR (for example, [77]); previously, we have already successfully applied this approach to the analysis of garlic gene expression [55,56,57]. The results were statistically analyzed with GraphPad Prism version 8 (GraphPad Software Inc., San Diego, CA, USA; https://www.graphpad.com/scientific-software/prism/ [accessed on 10 January 2023]). The data were expressed as the mean ± standard error (SE) based on three technical replicates of two biological replicates for each combination of cDNA and primer pairs. The unequal variance (Welch’s) *t*-test was applied to assess differences in gene expression; *p* < 0.01 was considered to indicate statistical significance.

### 4.5. Gene Amplification and Sequencing

To amplify the *AsSWEET* CDSs, gene-specific primers were designed based on *A. sativum* cv. Ershuizao transcriptomic data (NCBI project accession number: PRJNA607255) (Supplementary Table S2). PCR amplification was performed with 30 ng cDNA from the roots of each cultivar at the following conditions: initial denaturation at 95 °C for 5 min, 35 cycles of denaturation at 95 °C for 30 s, annealing at 55 °C for 30 s, and extension at 72 °C for 2 min, and final extension at 72 °C for 5 min. PCR products of the expected size were purified by using the QIAEX^®^ II Gel Extraction kit (QIAGEN, Hilden, Germany), cloned in the pGEM^®^-T Easy vector (Promega, Madison, WI, USA), and sequenced (3–5 clones for each accession) on ABI Prism 3730 DNA Sequencer (Applied Biosystems, Waltham, MA, USA) using the same primers.

### 4.6. Promoter Analysis

The search for *cis*-elements in the promoters (~1.0 kb regions upstream of the initiation codon) was performed using the PlantCARE database of *cis*-regulatory elements, enhancers, and repressors; (http://bioinformatics.psb.ugent.be/webtools/plantcare/html/; accessed on 26 January 2023). Promoter sequences and defining of hormone- and stress-responsive *cis*-elements are provided in Supplementary Tables S3 and S4, respectively.

### 4.7. Sucrose, Glucose, and Fructose Contents

About 0.2 g of freeze-dried ground roots was extracted twice with 200 µL of 80% methanol; the obtained extracts were pooled, evaporated, re-dissolved in 30% methanol to the final concentration of 50 mg fresh weight per 100 µL extract, and subjected to mass spectral analysis using ultra-performance liquid chromatography-quadrupole time-of-flight tandem mass spectrometry (UPLC-QTOF-MS/MS) according to [https://lcms.cz/labrulez-bucket-strapi-h3hsga3/1866243_lcms_148_how_potato_fights_its_enemies_02_2019_ebook_rev_01_9d3990d6c4/1866243-lcms-148-how-potato-fights-its-enemies-02-2019-ebook-rev-01.pdf; accessed on 1 December 2022].

## 5. Conclusions

We identified and characterized 27 genes encoding sugar uniporters of the SWEET family in the *A. sativum* cv. Ershuizao genome and cloned *AsSWEET3*, *AsSWEET9*, *AsSWEET11*, and *AsSWEET26* CDSs and promoter regions from FBR-resistant and -susceptible garlic cultivars. Structural analysis of *AsSWEET* genes indicated conserved functional involvement in the bidirectional transport of soluble sugars: hexoses (clade I *AsSWEET1*, *AsSWEET15*, *AsSWEET17*, *AsSWEET26*, and *AsSWEET27* and clade II *AsSWEET10*, and *AsSWEET12–14*), fructose (clade IV *AsSWEET20–25*), and sucrose (clade III *AsSWEET2–9*, *AsSWEET11*, *AsSWEET16*, *AsSWEET18*, and *AsSWEET19*). The *AsSWEET* promoters contained hormone- and stress-related elements associated with the response to fungal pathogens. Comparative *AsSWEET* transcriptional profiling in the *F. proliferatum*-infected roots of FBR-resistant and -susceptible cultivars suggests that clade III *AsSWEET3*, *AsSWEET9*, and *AsSWEET11* genes could be involved in the garlic response to *Fusarium* infection. Our results provide the first insights into the functions of *SWEET* genes in the plants of the *Allium* genus and may be used in breeding programs to increase the resistance of *Allium* crops to *Fusarium* infections.

## Figures and Tables

**Table 1:** Characteristics of the *SWEET* genes identified in the genome of *A. sativum* cv. Ershuizao.

Gene	Gene/Transcript ID [31]	Localization	Length, bp	Number of Exons	CDS, bp	Protein, aa	MW, kDa	pI	GRAVY	MtN3_slv Domain	TM Helix
*AsSWEET1*	Asa1G04426.1/Asa2G00182.1	ch1: 1194647983–1194654538 (−)	6556	7	699	232	25.998	9.18	0.607	15–99, 138–223	7
*AsSWEET2*	Asa2G01431.1/Asa3G01319.1	ch2: 399216486–399218187 (+)	1702	5	741	246	27.647	8.95	0.628	12–89, 123–205	7
*AsSWEET3*	Asa2G02061.1/Asa3G02137.1	ch2: 572338389–572339990 (−)	1602	6	834	277	31.033	9.43	0.666	12–99, 133–215	7
*AsSWEET4*	Asa2G05047.1/Asa3G05154.1	ch2: 1381249160–1381250776 (−)	1617	6	792	263	29.748	9.12	0.759	12–99, 133–215	7
*AsSWEET5*	Asa2G05048.1/Asa3G05155.1	ch2: 1382002246–1382003909 (+)	1664	6	774	257	29.044	9.37	0.748	12–99, 133–216	7
*AsSWEET6*	Asa2G05049.1/Asa3G05156.1	ch2: 1382472847–1382474148 (+)	1302	5	825	274	30.711	8.55	0.604	12–98, 133–215	7
*AsSWEET7*	Asa2G05056.1/Asa3G05153.1	ch2: 1383831994–1383833643 (+)	1650	6	792	263	29.754	9.20	0.698	12–99, 133–215	7
*AsSWEET8*	Asa2G05057.1/Asa3G05172.1	ch2: 1383951410–1383953045 (+)	1636	6	792	263	29.750	9.23	0.749	12–99, 133–215	7
*AsSWEET9*	Asa2G05060.1/Asa3G05167.1	ch2: 1384535061–1384536711 (+)	1651	6	789	262	29.544	9.26	0.714	12–99, 133–215	7
*AsSWEET10*	Asa2G07032.1/Asa3G07188.1	ch2: 1914150050–1914153509 (+)	3460	5	744	247	27.098	9.35	0.972	11–98, 134–218	7
*AsSWEET11*	Asa3G02607.1/Asa8G00525.1	ch3: 721990358–721991933 (+)	1576	6	819	272	30.456	9.30	0.696	12–99, 133–215	7
*AsSWEET12*	Asa3G03715.1/Asa8G02144.1	ch3: 1020592700–1020594065 (−)	1366	5	762	253	28.012	8.89	0.860	11–94, 132–218	7
*AsSWEET13*	Asa5G00452.1/Asa6G00626.1	ch5: 108642296–108644275 (−)	1980	3	654	217	23.991	9.36	0.835	1–65, 103–189	6
*AsSWEET14*	Asa5G01067.1/Asa6G01212.1	ch5: 246733367–246737173 (−)	3807	6	1002	333	37.185	9.75	0.203	116–183, 220–301	6
*AsSWEET15*	Asa5G05357.1/Asa5G00822.1	ch5: 1446881843–1446885138 (−)	3296	5	813	270	29.239	9.36	0.552	40–122, 159–241	6
*AsSWEET16*	Asa6G00924.1/Asa6G07296.1	ch6: 237675722–237676674 (+)	953	4	699	232	25.961	5.84	0.613	1–42, 76–162	5
*AsSWEET17*	Asa7G01759.1/Asa1G02255.1	ch7: 480861997–480863949 (+)	1953	6	735	244	27.281	9.28	0.644	7–95, 129–214	7
*AsSWEET18*	Asa7G03377.1/Asa1G00572.1	ch7: 914393920–914395735 (−)	1816	6	777	258	29.040	9.07	0.796	12–99, 133–214	7
*AsSWEET19*	Asa7G03379.1/Asa1G00574.1	ch7: 914587432–914589248 (+)	1817	6	777	258	29.040	9.07	0.796	12–99, 133–215	7
*AsSWEET20*	Asa8G00607.1/Asa6G05670.1	ch8: 193855534–193857365 (+)	1832	6	864	287	31.852	9.54	0.515	6–93, 129–212	7
*AsSWEET21*	Asa8G00611.1/Asa7G00384.1	ch8: 194959619–194961439 (+)	1821	6	849	282	31.369	10.01	0.612	6–93, 129–212	7
*AsSWEET22*	Asa8G00624.1/Asa7G00398.1	ch8: 197356527–197358249 (−)	1722	6	705	234	25.832	9.68	0.765	6–93, 129–210	7
*AsSWEET23*	Asa8G00626.1/Asa7G00406.1	ch8: 197411881–197415626 (−)	3746	7	936	311	34.077	8.87	0.797	6–91, 127–208	6
*AsSWEET24*	Asa8G00629.1/Asa8G00420.1	ch8: 198119328–198122294 (−)	2967	6	840	279	30.696	6.82	0.778	25–111, 157–233	6
*AsSWEET25*	Asa8G00631.1/Asa7G00403.1	ch8: 198345649–198347027 (+)	1379	6	705	234	25.907	9.58	0.795	6–93, 129–213	7
*AsSWEET26*	Asa8G02736.1/Asa7G02662.1	ch8: 756634184–756635906 (−)	1723	6	711	236	26.380	8.79	0.852	19–105, 139–224	7
*AsSWEET27*	Asa0G02772.1/Asa8G01762.1	scaffold24049: 67334–68424 (−)	1091	5	720	239	26.761	8.19	0.741	21–102, 141–225	7

MW, molecular weight; pI, isoelectric point; GRAVY, grand average hydropathy; CDS, coding sequence; MtN3_slv, superfamily of sugar efflux transporters for intercellular exchange; TM, transmembrane.

**Table 2:** Polymorphisms in the *AsSWEET* coding sequences and regulatory regions in cv. Sarmat and Strelets compared to cv. Ershuizao.

Gene	NCBI ID (Sarmat/Strelets)	SNP (aa Substitution) in CDS		SNPs and Indels in Regulatory Region	
		cv. Sarmat	cv. Strelets	cv. Sarmat	cv. Strelets
*AsSWEET3*	OQ607029/OQ607030	c. 282T>C, **c. 293A>G (p. K98R)**, c. 351G>A, c. 387T>C, c. 711T>C		-768A>T, -769A>G, -778C>T, del. ˗239.. ˗579 (340 bp), insertion 3 bp (˗704)	**˗188 T>A**, ˗768A>T, ˗769A>G, ˗778C>T, **˗832T>C**, del. ˗239.. ˗579 (340 bp)
		**c. 758T>C (p. V253A)**			
*AsSWEET9*	OQ607031/OQ607032	c. 153C>T, c. 336A>T, c. 582G>A		˗307G>A, ˗411C>T, ˗412T>C, ˗419A>G, ˗429C>T, ˗450T>G, ˗454A>G, ˗456C>G, ˗459A>T, ˗475A>G, ˗910C>T, del. ˗595.. ˗596 (2 bp)	˗307G>A, ˗411C>T, ˗412T>C, ˗419A>G, ˗429C>T, ˗450T>G, ˗454A>G, ˗456C>G, ˗459A>T, ˗475A>G, ˗910C>T, del. ˗595.. ˗596 (2 bp)
*AsSWEET11*	OQ607033/OQ607034	c. 72C>T, c. 75T>C, c. 324G>A, c. 813C>A, c. 734A>T		˗501C>T, ˗551T>C	˗501C>T, ˗551T>C
		**c. 734A>T (p. E245V)**	c. 69C>T, **c. 761T>C (p. I254T)**		
*AsSWEET26*	OQ607035/OQ607036	c. 204C>A, c. 534T>C		**˗225G>A**, **˗291T>A**, ˗515T>G, **˗528C>T**, **˗564G>A**, ˗578A>G, ˗596T>G, ˗671G>T, ˗673T>C, ˗791T>A, ˗849T>C, ˗856T>C, **del. ˗624.. ˗633 (10 bp)**	**˗222T>C**, **˗259G>A**, **˗355A>G**, ˗515T>G, **˗516T>C**, **˗533T>A**, **˗539T>G**, ˗578A>G, **˗591G>A**, ˗596T>G, **˗604A>C**, **˗653A>G**, **˗667T>C**, ˗671G>T, ˗673T>C, ˗791T>A, ˗849T>C, ˗856T>C

Note: Non-synonymous SNPs in CDS and corresponding amino acid substitutions, as well as cultivar-specific polymorphisms in gene regulatory regions are marked in bold.

**Table 3:** Hormone- and stress-related *cis*-acting elements found in the upstream region of *AsSWEET3*, *9*, *11*, and *26* genes.

	Elements	*AsSWEET3*			*AsSWEET9*			*AsSWEET11*			*AsSWEET26*		
		Ershuizao	Sarmat	Strelets	Ershuizao	Sarmat	Strelets	Ershuizao	Sarmat	Strelets	Ershuizao	Sarmat	Strelets
Hormone-related	ABRE										1	1	1
	AuxRR-core										1		1
	TGA-element											1	1
	CGTCA-motif	1	1	1	1	1	1					1	1
	TCA-element	2	2	2	1	1	1						
	ERE	1	1	1									
	P-box											1	1
Stress-related	ARE	1	1	1	4	4	4				2	1	1
	STRE	1						2	1	1	1	1	1
	LTR				1	1	1						
	WRE3			1							1	1	1
	WUN-motif				1	1	1						
	GC-motif							2	2	2			
Other	AAGAA-motif	1	1	1	1	1	1	1	1	1	1	1	1
	Box 4	1	1	1		1	1				1	1	1
	F-box	1											
	AE-box				3	2	2	1	1	1			
	CAT-box				1	1	1						
	G-box				1	1	1				2	2	2
	ATC-motif							1	1	1			
	TCT-motif												
	Circadian							1	1	1			
	AT-rich element										1	1	1
	CTAG-motif										1	1	1
	O2-site										1	1	1
	Box S										1	1	1
	MYC	3	3	3				3	3	3	1	2	2
	MYB				2	2	2				3	1	2

## Data Availability

*AsSWEET* CDSs of *A. sativum* cv. Sarmat/Strelets are available at NCBI (https://www.ncbi.nlm.nih.gov/ accessed on 1 March 2023) (see Table 2).

## Supplementary Materials

The following supporting information can be downloaded at: https://www.mdpi.com/article/10.3390/ijms24087533/s1.
